# MiRNAs in Gestational Diabetes Mellitus: Potential Mechanisms and Clinical Applications

**DOI:** 10.1155/2021/4632745

**Published:** 2021-11-24

**Authors:** Zhao-Nan Liu, Ying Jiang, Xuan-Qi Liu, Meng-Meng Yang, Cheng Chen, Bai-Hui Zhao, He-Feng Huang, Qiong Luo

**Affiliations:** ^1^Department of Reproductive Genetics, Women's Hospital, School of Medicine, Zhejiang University, Hangzhou 310006, China; ^2^Department of Obstetrics, Women's Hospital, School of Medicine, Zhejiang University, China

## Abstract

Gestational diabetes mellitus (GDM) is a common pregnancy complication which is normally diagnosed in the second trimester of gestation. With an increasing incidence, GDM poses a significant threat to maternal and offspring health. Therefore, we need a deeper understanding of GDM pathophysiology and novel investigation on the diagnosis and treatment for GDM. MicroRNAs (miRNAs), a class of endogenic small noncoding RNAs with a length of approximately 19-24 nucleotides, have been reported to exert their function in gene expression by binding to proteins or being enclosed in membranous vesicles, such as exosomes. Studies have investigated the roles of miRNAs in the pathophysiological mechanism of GDM and their potential as noninvasive biological candidates for the management of GDM, including diagnosis and treatment. This review is aimed at summarizing the pathophysiological significance of miRNAs in GDM development and their potential function in GDM clinical diagnosis and therapeutic approach. In this review, we summarized an integrated expressional profile and the pathophysiological significance of placental exosomes and associated miRNAs, as well as other plasma miRNAs such as exo-AT. Furthermore, we also discussed the practical application of exosomes in GDM postpartum outcomes and the potential function of several miRNAs as therapeutic target in the GDM pathological pathway, thus providing a novel clinical insight of these biological signatures into GDM therapeutic approach.

## 1. Overview of Gestational Diabetes Mellitus

Gestational diabetes mellitus (GDM) is a common maternal complication that occurs or is recognized during pregnancy. Since the pathophysiology of GDM is characterized by chronic insulin resistance in the second half of pregnancy, it is not diagnosed until the late second or early third trimester of gestation. A globally estimated prevalence of 1.8-31% has been reported due to the lack of consistency in GDM diagnostic criteria between countries [1]. GDM exerts various adverse implications for mothers and their offspring. Mothers complicated by GDM have higher rates of preeclampsia and adverse pregnancy outcomes, such as cesarean deliveries and shoulder dystocia [2]. They are more susceptible to developing postpartum type 2 diabetes mellitus (T2DM) compared with normal women [3]. Additionally, their offspring may suffer from long-term metabolic disorders and related health conditions such as obesity, T2DM, and cardiovascular disease (CVD) [4, 5]. According to a new set of diagnostic criteria published by IADPSG, pregnant women should perform an oral glucose tolerance test (OGTT) during 24-28 weeks of gestation [6]. However, compliance with the test may decline because it requires fasting and multiple blood types and may cause discomfort such as vomiting. However, OGTT is not recommended as a routine screening for GDM at an earlier trimester of gestation [6], and hence, treatment cannot be applied promptly for the prevention of GDM. Therefore, finding early predictors of GDM is significant to improve the prognosis of mothers and fetuses.

Moreover, since coronavirus disease 2019 (COVID-19) pneumonia pandemic-induced local lockdown measures have been carried out worldwide, their negative effects on psychosocial states of pregnant women and on the glycemic balance in GDM patients have been observed. A review indicated negative implications of lockdowns and unhealthy lifestyle for pregnancy [7]. Solitude and mental burden such as anxiety and depression may lead to unhealthy dietary habits and reduced exercise. On the other hand, increased snack consumption and carbohydrate intake were revealed with a high glycemic index; increased total diet intake was found to be associated with a rise in HbA1c levels [8, 9] during the COVID-19 pandemic lockdown. A retrospective study conducted in France reported a lower postprandial glycemic control and a higher use of insulin therapy during quarantine (18 March–7 May 2020). These observations were explained by anxiety, reduced physical activity, and changes in diet [10]. These risk factors, coordinating with self-reported boredom/solitude and enhanced consumption of snacks, unhealthy foods, and sweets, have caused increased weight gain in some obese individuals [11]. A higher rate of GDM was observed in pregnant women during March–April 2020 compared with the same period in 2019 [12].

miRNAs, first discovered from C. elegans by Ambros and Ruvkun, represent small, short noncoding, and single-stranded RNA sequences consisting of approximately 22 nucleotides (nt) in length and act as negative regulators by inhibiting mRNA translation or leading to its degradation [13]. In most cases, miRNAs can also mediate posttranscriptional gene silencing by complementary binding to the target mRNA 3′-untranslated region (3′-UTR) or 5′-UTR or open reading frame (ORF) regions via their seed sequence region [14, 15]. Many animal model systems have been established to detect miRNAs, and their number is primarily associated with the organism's complexity [16]. miRNAs present potential roles in the regulation of *β*-cell function and mass, as well as in metabolic processes [17]. The genome-wide analysis has demonstrated over 600 miRNAs expressed in placenta and their essential role in pregnancy and GDM [17–19]. Given the high stability of placental miRNAs in maternal circulation and their accessibility from maternal blood, they may become an early diagnostic biomarker of GDM [19]. Meanwhile, the role of a low glycemic or Mediterranean diet and particularly the favorable impact of plant-derived foods (e.g., vegetables, fibers, and fruits) on oxidative stress by enhancing antioxidant compounds has represented a new aspect in the pathogenesis of GDM [20]. Moreover, the correlation with miRNAs was not fully understood. This review is aimed at reporting updated literature in miRNA regarding to pathogenesis of GDM and the associated potential application.

## 2. The Biogenesis Pathway for miRNAs

**Figure 1:**
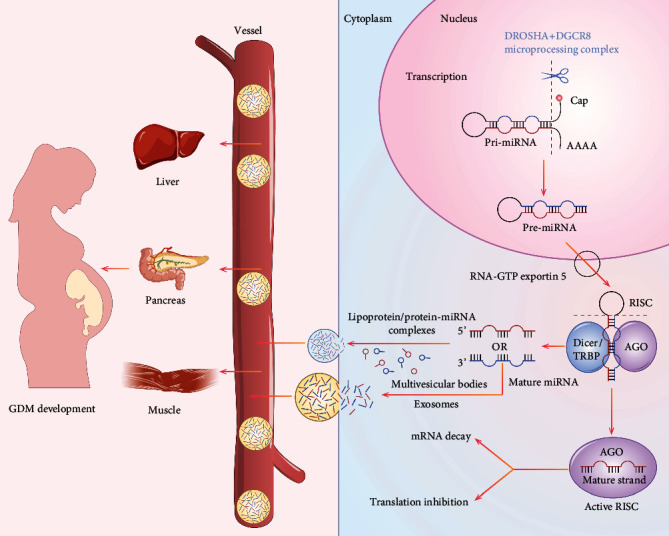
Overview of the biogenesis and potential functions of miRNAs in GDM. The biogenesis pathway for miRNAs is shown on the right side: pri-miRNA is cropped by the microprocessor complex to produce a pre-miRNA, which is then exported to the cytoplasm by the Exportin5-RanGTP system. The Dicer/TRBP complex cleaves the terminal loop of the pre-miRNA to create a miRNA duplex [21]. The latter is loaded into the RISC and further unwinds in the Argonaute (AGO) protein, the center of RISC [21, 22]. During this process, the guide RNA strand from the miRNA duplex is selected as the mature miRNA, while the other passenger RNA strand is degraded. This guide strand remains in the RISC to form the miRNA-RISC complex as an essential component [23], serving to play a role in mRNA decay or translation inhibition [13, 24–28]. Furthermore, miRNA is exported to the extracellular space through various carriers such as lipoproteins, proteins, and exosomes [34]. The participation of miRNA in the pathogenesis of GDM is shown on the left: these miRNA complexes are transported in the vessel and exert their potential functions on the pathogenesis of GDM through tissues/organs that linked to glucose metabolism (e.g., liver, pancreas, and muscle), serving as diagnostic biomarkers and therapeutic target in GDM.

During the process of miRNA biogenesis (shown in Figure 1), miRNAs located in intergenic regions and introns are transcribed by RNA polymerases II and III, from their promoter or cotranscribed with their own host gene or other miRNAs in the initial stage. The primary miRNA (pri-miRNA), an ~1000 nt capped and polyadenylated transcript, is known to contain a stem-loop structure in the nucleus [21]. The microprocessor complex subsequently crops this pri-miRNA to produce a precursor miRNA (pre-miRNA) with a length of 60 nt. The Exportin5-RanGTP system then exports this pre-miRNA to the cytoplasm for further processing. Eventually, the Dicer/TRBP complex cleaves the terminal loop of the pre-miRNA to create a miRNA duplex [21].

The remaining double-stranded RNA is loaded into a multiprotein complex called an RNA-induced silencing complex (RISC) and further unwinds in the center of RISC (an Argonaute protein) [21, 22]. During this process, the guide RNA strand from the miRNA duplex is selected as the mature miRNA, while the other passenger RNA strand is degraded. This guide strand remains in the RISC to form the miRNA-RISC complex as an essential component and serves to regulate gene expression epigenetically [23].

The miRNA-RISC has the capacity to regulate gene expression through base-pairing to the 3′-untranslated region (UTR), 5′-UTR, and protein-coding region of the messenger RNA (mRNA) target [13, 24]. The specific interaction between miRNAs and the target mRNA is primarily directed by the miRNA binding. This binding requires a certain number of nucleotides to match the sequence flanking the seed region [25]. The processes of the regulation in gene translation by miRNA-RISC are divided into two steps [26]: (i) the miRNA-RISC complex obstructs the binding between ribosomes and the mRNA target [27]; (ii) this consequently leads to mRNA degradation characterized by mRNA deadenylation and decapping, leading to accelerated destabilization and decay, thus suppressing translation of the target mRNA ultimately [28].

A single miRNA can target hundreds of mRNAs, and a specific target mRNA is often under the control of several distinct miRNAs. It has been established that miRNAs have potential function in many essential biological activities, such as cell proliferation, differentiation, apoptosis, disease initiation, and development [29–33]. Their dysregulation or dysfunction was revealed in many metabolic researches regarding obesity, T2DM, and cardiovascular disease. In addition, extracellular miRNAs are present in biological fluids such as plasma and are being packed into various carriers such as microvesicles (e.g. exosomes) or lipoproteins, rendering them a potential role as biomarkers or therapeutic targets [34].

## 3. miRNA Identification and Quantification Techniques

Several specific and sensitive approaches were applied to detect, validate, and quantify miRNAs, including quantitative reverse transcriptase PCR (RT-qPCR) [35, 36], in situ hybridization [37], Northern blot analysis [38, 39], miRNA microarray [40, 41], and next-generation sequencing(NGS) [42]. Deciding on the optimal miRNA profiling and quantification technology depends on the experimental designs, specific types of sample, research objective, and intended therapeutic use.

However, the expressions of several miRNAs in some findings we will review in detail further are not shared across each other. Different source materials such as serum or plasma used during the detecting process or discrepancy in the analysis platform's application might contribute to such differences [43]. Therefore, minimizing experimental variations through experimental normalization, data processing, and optimization is also significant for the precise evaluation of the level of miRNA from a specific sample [44].

Currently, quantitative reverse transcriptase PCR (RT-qPCR) is known as the gold-standard approach for miRNA quantification, which serves as the most reproducible and sensitive method [45–47]. Stem-loop RT-based TaqMan miRNA assay is widely used as the main PCR technique in research due to the advantage of high sensitivity and specificity [48]. Besides, direct RT-based and poly(A) tailing-based SYBR miRNA assays are considered as practical alternatives for miRNA detection and quantification [48]. A high-throughput qRT-PCR platform has been established as a more available approach for rapid miRNA profiling of a great quantity of biological samples. Some advances have been achieved in quantification using low amounts of miRNA [49]. TaqMan low density array (TLDA) possesses the advantage of cost-effectivity and serves as the most widely used qRT-PCR miRNA expression profiling method [50].

Efforts have been made for the possibility of shortening the technique execution time as well as lowering amounts of miRNA used in quantification [51–53]. Microarrays represent a practicable discovery tool used for miRNA identification on the basis of the principle in hybridization of cDNA to the DNA probe [54]. However, this technique is not quite promising for miRNA profiling, since they are not capable of detecting highly expressed miRNAs or distinguishing between mature and immature miRNAs [55, 56]. Moreover, several limitations related to this technique including low sensitivity, high requirement for RNA input amount (100 ng to 1 *μ*g), background, and cross-hybridization still remain to be solved.

Another two alternatives for miRNA identification are also applied in research. In situ hybridization serves to contrast the level of miRNAs in different cells through utilizing radioactive, fluorescent, or dioxygen in probes [57]. It is noted that ISH also presents several disadvantages, including long processes, strenuous steps, and a higher rate of errors [57]. Additionally, next-generation sequencing technology (NGS) is a highly accurate technique with an advantage over other technologies, as it has the capability to identify novel miRNAs. Nevertheless, NGS is a more laborsome technique compared with qRT-PCR and microarrays and presents a higher requirement for RNA input amount (500 ng to 5 *μ*g). Of note, high costs of this technique may contribute to a limitation of its wider availability [58].

Moreover, the most frequent normalization technique involves strategies using exogenous spike-in miRNA, such as C. elegans miR-39, which is validated to be more reliable compared with endogenous reference genes like miR-16 [59]. However, researchers prefer an application of combining both exogenous and endogenous miRNA reference genes, due to no ideal normalization strategy exists and the application of a single type of reference gene is insufficient for accurate miRNA results [60].

## 4. miRNA: The Role in Pathophysiology of GDM

### 4.1. miRNA-Related Maternal Metabolic Adaptation

In the past decade, people have been interested in the link of novel placenta-derived factors such as placenta-derived miRNA to pregnancy. More and more studies have explored the biological functions of placental-derived miRNA and their applicability as biomarkers in some pregnancy complications, such as GDM. Moreover, it is well established that an improper maternal metabolic adaptation to these placental-derived miRNAs has been observed [61, 62]. Therefore, variations in the expression of placental-related miRNA may indicate changes to maternal metabolic adaptation mechanism, thus providing insight into the pathogenesis of GDM pregnancy. Besides, variations in the expression of miRNAs in circulating samples may also indicate their involvement in maternal metabolic adaptation. Several studies have investigated the regulation of placenta-associated miRNA and circulating miRNAs as well as their related metabolic adaptation in GDM (Table 1).

Kokkinopoulou et al. first described a T2DM-specific expression profile of miRNAs that target disease-susceptibility genes, such as CDKN2A, CDK5, IGF2BP2, KCNQ1, and TSPAN8. miR-98-5p, one of miRNAs expressing decreased levels in T2DM patients compared with controls, was reported [63]. Moreover, miR-98 is also known to be implicated in embryo implantation during the initial stage of pregnancy. In 2016, Cao et al. showed a significant upregulation of miR-98 derived from placenta at gestation of 37–40 weeks in GDM patients (*n* = 193) compared to normal pregnant subjects (*n* = 202). Additionally, experimental validation in JEG-3 (human choriocarcinoma cell line) provided supportive evidence for its role in the regulation of glucose uptake. Specifically, by regulating Mecp2 and in turn targeting Trpc3, it has subsequent regulative effects on insulin-mediated glucose uptake in GDM [64]. This experimental evidence further confirmed the role of miR-98 in the development of GDM.

Zhao et al. reported a significantly upregulated concentration of miR-518d in the placenta of women affected by GDM compared with the normal subjects at 37-40 weeks of gestation. It is further proven that concentration of miR-518d in term placenta was negatively correlated with the expression of peroxisome proliferator-activated receptor-*α* (PPAR*α*) [65]. PPAR plays a role in regulating the pathway related to inflammation, accidental formation, oxidative stress, and insulin signaling metabolism [66, 67]. The downregulation of the PPAR*γ* expression in GDM may accelerate glucose intolerance [68]. Reduced expression of PPAR*α* and RXR*α* were also found in the placenta of women with GDM [69].

In 2011, the same group demonstrated a significant downregulation of miR-132, miR-29a, and miR-222 in serum derived from GDM patients (*n* = 24) at gestation during the 16th and 19th weeks in comparison with healthy pregnant women (*n* = 24) [50]. Contrarily, a significant upregulation of miR-222, 1 of 17 differentially expressed miRNAs identified by Shi et al., was found in omental adipose tissues from GDM patients. By conducting a validation study in 10 GDM pregnant women compared with 10 healthy subjects of normal glucose tolerance, they further confirmed that the level of miR-222 was negatively correlated with the protein concentration of transporter glucose transporter 4 (GLUT4) in omental adipose tissue, as well as estrogen receptor- (ER-) *α*; the implication of the latter was validated in glucose homeostasis and insulin regulation [70–72]. Furthermore, they also validated the involvement of miR-222 in insulin resistance induced by estrogen in GDM through experiments performed on 3T3-L1 adipocytes by using antisense oligonucleotides [55].

Later on, Stirm et al. demonstrated a significant upregulation of miR-340 in whole blood cells (WBC) and lymphocytes from GDM women (*n* = 8) at 24–32 weeks of gestation, compared to healthy subjects (*n* = 8) [73]. A significant downregulation of polyadenylate- (poly(A)-) binding protein- (PABP-) interacting protein 1 (PAIP1), known as a key promoter of translation that was never described in GDM before [74], was observed only in WBCs in GDM women, in comparison with normal glucose tolerant (NGT) subjects. An inverse correlation between miR-340 and PAIP1 expression in lymphocytes was observed, indicating that miR-340 might negatively regulate PAIP1. They further conducted experiments and observed reduced expression of miR-340 in human lymphocytes cultured in high-glucose medium. After adding insulin to the high-glucose medium, the miR-340 level presented an inversely significant increase, indicating that miR-340 expression was regulated in a context of insulin resistance. Accordingly, the expression of miR-340 in leukocytes is positively correlated with the level of maternal fasting insulin in vivo.

Finally, Tryggestad et al. identified differentially expressed miRNAs by using a miRNA microarray in HUVECs from GDM-exposed newborns (*n* = 7) with respect to normal newborns (*n* = 12) [75]. Seven upregulated miRNAs were found in HUVECs from GDM-exposed newborns and selected for validation by RT-qPCR, including miR-130b-3p and miR-148a-3p. They also observed the reduced expression of AMP-activated protein kinase *α*1 subunit (AMPK*α*1) in GDM-exposed placenta. Notably, miR-130b and miR-148a were validated to posttranscriptionally regulate AMPK*α*1 [75]. AMPK*α*1 is known to be involved in regulation of genes related to energy homeostasis, fatty acid synthesis, protein synthesis, and glucose metabolism by functioning as a central enzyme [76]. Recent data have also demonstrated that AMPK, whose activity is significantly reduced in adipose tissue and skeletal muscle of GDM women, was downregulated in placenta of pharmacologically treated GDM patients [77, 78]. Furthermore, pAMPK was confirmed to activate the mTOR pathway and contribute to the conversion toward aerobic glycolysis in GDM.

### 4.2. miRNA-Related Maternal Pancreatic *β*-Cell Dysfunction

The development of GDM may be attributed to the dysfunction of maternal pancreatic *β*-cell during the compensatory mechanism for insulin resistance. Recent studies have established a conceivable link between circulating miRNAs, placental miRNAs, and maternal pancreatic *β*-cell dysfunction in GDM (Table 2).

Feng et al. assessed the level of miRNAs in peripheral blood samples derived from 12 GDM pregnancies and 12 healthy pregnancies. miR-33a-5p was demonstrated to be significantly upregulated in GDM group with respect to the NGT group. Furthermore, the authors found a positive correlation between miR-33a-5p expression and blood glucose. Notably, overexpression or inhibition of miR-33a-5p performed on INS-1 cells was revealed to significantly inhibit or promote cell growth and insulin production under high glucose condition, respectively. miR-33a-5p was found to directly target its downstream gene ABCA1, and lnc-DANCR exerts as a sponge in the regulation of antagonizing the function of miR-33a-5p [79]. These results confirmed that the lnc-DANCR-miR-33a-5p-ABCA1 signaling pathway exerts a significant role in regulating the biological function of INS-1 cells.

Similarly, Sebastiani et al. evaluated the level of miR-330-3p and found its hyperexpression in the blood sample of 21 GDM pregnancies versus 10 normal pregnancies at 24–33 weeks of pregnancy using a highly standardized approach. Interestingly, circulating miR-330-3p expression was negatively associated with fasting insulin only in GDM patients. Furthermore, two age- and BMI-matched populations were distinguished by differential level of miR-330-3p that divided into high and low groups, respectively [80]. Moreover, overexpression of miR-330-3p was validated to target and downregulate key genes, such as E2F1, known as essential modulators in glucose-stimulated insulin secretion and *β*-cell maintenance, such as *β*-cell growth and proliferation [81, 82]. The authors thus postulated that the hyperexpression of miR-330-3p in the blood sample may be harmful for *β*-cell function and/or proliferation.

Oppositely, He et al. analyzed the expression of miR-494 in the blood sample from 20 pregnancies affected by GDM and 20 normal women [83]. A significant downregulation of miR-494 was found in GDM pregnancies compared to CTRLs and was negatively associated with blood glucose. Furthermore, overexpression of miR-494 enhanced insulin secretion, induced cell proliferation, and inhibited cell apoptosis, whereas miR-494 knockdown achieved the opposite results. miR-494 was revealed to directly target phosphatase and tensin homolog (PTEN), known to exert a crucial role in apoptosis, in pancreatic *β*-cells. Notably, downregulation of PTEN induced by siRNA rescued the impact brought by miR-494 knockdown on insulin secretion, cell proliferation, and apoptosis of pancreatic *β*-cells. In conclusion, the results underline implication of miR-494 in *β*-cell dysfunction of GDM.

Li et al. also reported a significant downregulation of miR-96 in placental tissue from 3 GDM pregnancies compared to 3 healthy pregnancies. In addition, miR-96 expression was also found inversely correlated with blood glucose. It is noted that the knockdown of miR-96 reduced insulin level, lowered cell viability, and increased apoptosis in INS-1 cells under high glucose condition. Interestingly, similar correlation between miRNA and blood glucose was also observed in GDM rats. Zhao et al. analyzed the miRNA-221 expression in placental tissues of GDM rats by the microarray. A downregulation of miRNA-221 was reported in GDM rats, and a negative correlation between the miRNA-221 level and the blood glucose level was demonstrated. Notably, knockdown of miRNA-221 lowered insulin production and increased apoptosis in INS-1 cells, while opposite results were observed in miRNA-221-overexpressed INS-1 cells. Of note, miRNA-221 and miR-96 were proven to directly target PAK1 in two researches, and these results suggested that the dysfunction of *β*-cell might be attributed to dysregulation of miRNA-221 and miR-96 with a subsequent effect through targeting PAK1 [84, 85].

## 5. miRNAs in Placental Function and Fetal Complication

We have reviewed the role of several placenta-associated and circulating miRNAs in maternal metabolic adaptation and pancreatic *β*-cell dysfunction. In addition, several studies have investigated the role of miRNAs in placental function, as well as GDM-related fetal complication of the next generation.

By using RNA sequence and qRT-PCR validation, Ding et al. confirmed several dysregulated miRNAs in the placenta derived from 8 GDM pregnancies versus 8 healthy subjects. These differentially expressed miRNAs were predicted to be involved in placenta morphology and development. Notably, miR-138-5p was selected for biological functional assay due to its significant overexpression in GDM. Its overexpression inhibited the proliferative and migration ability of HTR-8/SVneo trophoblast cells. A specific target of miR-138-5p was TBL1X, an oncogene in the activation of the WNT/*β*-catenin signaling pathway. This pathway crucially participates in placental biological processes, such as proliferation, differentiation, and invasion [86–88]. Moreover, miR-138-5p was validated to target sirtuin 1 (SIRT1) [89]; although limited studies reported the association between SIRT1 and GDM, reliable data confirmed its implication in the inflammation and glucose metabolic pathway in human placenta. Mac-Marcjanek et al. conducted experiments to investigate SIRT1-dependent specific gene alteration in GDM pregnancies and identified four diabetes-relevant genes linked to metabolism, inflammation, and transporting functions in SIRT1-overexpressed leukocytes [90]. SIRT1 was also found increasingly expressed in GDM women exposed to hyperglycemia at one day postpartum [91]. However, other authors observed a reduced level of SIRT1 in fetal endothelial colony-forming cells (ECFCs) and HUVECs in GDM pregnancies [92, 93], suggesting that dysregulation of SIRTs may be related to fetal complication. These evidences suggested that miR-138-5p serves as a potential biomarker in GDM management.

Li et al. identified 29 differentially expressed placenta-derived miRNAs from 15 GDM pregnancies in respect to 15 normoglycemia subjects to investigate the alteration of miRNAs. By conducting a miRNA microarray and RT-qPCR analysis approach, they validated 9 dysregulated miRNAs (miR-508-3p, miR-27a, miR-9, miR-137, miR-92a, miR-33a, miR-30d, miR-362-5p, and miR-502-5p). Furthermore, these miRNAs were predicted to target key genes implicated in the EGFR/phosphatidylinositol 3-kinase (PI3K)/protein kinase B (AKT) signaling pathway [56]. Of note, it is well known that the insulin tyrosine kinase receptor could activate the PI3K/AKT pathway and promote glucose transporting by enhancing the delivery of intracellular GLUT4 to the cell surface. Specifically, miR-508-3p, one of the overexpressed miRNAs, was revealed to directly regulate PIKfyve, a reverse modulator of the epidermal growth factor receptor (EGFR). PIKfyve exerts an essential role in adequate placental development and fetal growth [94]. The upregulation of miR-508-3p was validated to repress the expression of PIKfyve and aberrantly activate the EGFR/PI3K/AKT signaling [56]. Thus, the dysregulation of miR-508-3p may potentially promote the development of macrosomia, a specific fetal complication related to GDM.

Floris et al. reported an upregulated expression of miR-101 in HUVEC cells from GDM (*n* = 22) compared to healthy subjects (*n* = 24) and confirmed its crucial role in endothelial function and angiogenesis [95]. Moreover, miR-101 was found to target enhancer of zester homolog 2 (EZH2) [95–100], which exhibited reduced concentration in its isoform and histone H3K27 trimethylation in cultured human umbilical vein endothelial cells (HUVECs) from a GDM-exposed fetus [101]. A negative correlation between miR-101 and EZH2 was reported in a feedback loop of epigenetic regulation, suggesting a decreased functionality in GDM placenta. The dysfunctionality of the GDM placenta may contribute to miR-101 upregulation and functional alterations observed in HUVECs, including cell apoptotic activities and angiogenic and migratory capacities [101]. However, the maintenance of the alteration in this pathway and associated adverse impact on generation's health still remain unclear. Some metabolic disorders such as cardiovascular disease might emerge in their adulthood life.

Notably, miRNAs could also function as a protective mechanism. Diaz-Perez et al. discovered another two differentially expressed miRNAs in GDM placental tissue and revealed their potential role in placental pathophysiology. Specifically, upregulation of miR-221 and miR-222 was reported in human fetoplacental endothelial cells (fpEC) isolated from four GDM placentas during the third trimester compared to four CTRLs [102]. What is more, miR-221 and miR-222 were validated to negatively regulate ICAM1 protein, whose reduced concentration was observed in the fetoplacental endothelium derived from GDM [103, 104]. These miRNAs may lead to the downregulation of ICAM-1 and function as a protective mechanism against inflammation characterized by leucocyte transmigration from blood to placenta due to hyperglycemia during GDM [102].

## 6. Exosomes and miRNAs in GDM

Exosomes are known specifically as extracellular vesicles (EVs), with the characteristic of a bilayered lipid and ~50-150 nm in diameter, originating from the endosomal compartment and actively secreted by multiple cell types [105]. Recently, exosomal miRNAs and their involvement in gene expression are gaining increasing scientific attention, suggesting their potential role for regenerating new therapies [106]. Exosomal miRs can be derived from different biological fluids, such as saliva, serum, amniotic fluid, urine, and breast milk, and can be released from various cells into the extracellular space [107, 108]; such a characteristic renders them to be potential clinical biomarkers and even novel targets for therapeutic intervention.

Three modes of mechanisms have been reported in the protection of miRNAs from degradation [34, 109–113]. These mechanisms could guarantee intercellular communication of miRNAs and their stability as cargos when delivered to recipient cells, subsequently inducing expressional and functional response. Therefore, similar to the cell-to-cell contact-dependent signaling pattern, the capacity of circulating EVs in conveying information is also considered an essential way for intercellular communication [114].

It is widely acknowledged that placenta is tightly linked to alteration of metabolic status in pregnancy. It is considered that adverse placental condition might be mirrored by the miRNA expression profile in placenta-derived exosomes (PdEs). In this part, we will emphasize PdE's contribution to the development of GDM and give our viewpoints for their application in GDM management.

### 6.1. Tissue-Derived Exosome and Exosomal miRNAs in GDM

Rice et al. performed the pilot study to demonstrate an altered exosomal concentration in GDM pregnancy. They observed a significantly higher exosomal level in the plasma sample of GDM women compared to normal subjects. The results also revealed that a high D-glucose level promotes exosomes released from trophoblast cells during the first-trimester pregnancy, suggesting a correlation between high glucose and exosomal bioactivity, which is of clinical relevance in GDM pathophysiology [115]. Furthermore, these exosomes released from trophoblast cells were confirmed to induce the expression of cytokine mediators such as interleukin-8 (IL-8) and TNF-a by in vitro experiments conducted on human umbilical vein endothelial cells (HUVECs), suggesting that exosomes could regulate immune responses to maternal metabolic adaptation during pregnancy.

Another study conducted by Salomon et al. also investigated the profile of PdEs in plasma during pregnancy. A progressive increase in the amount of these PdEs was observed, and the profile of these PdEs released into peripheral circulation at the 6-week gestation was characterized by gestational age. Furthermore, Salomon et al. confirmed these results in a prospective cohort through comparing the gestational-age PdE profile in GDM maternal plasma to normal subjects [116]. Similarly, they also observed an altered release of proinflammatory cytokines from HUVECs when treated with these PdEs derived from GDM pregnant women [117]. A more recent study conducted by Nardi et al. also reported similar results, indicating such pregnancy-related alterations of circulating EVs might provide a first hint for their role in the regulation of immune response during pregnancy [118].

Nakahara et al. also reported total PdE exosomal alterations in a cohort study and revealed their association with gestational age and pregnancy outcome. They also found a significantly higher PdE level in GDM pregnancies and PE versus normal pregnancies. In addition, several significant risk factors for GDM, including glucose concentration, maternal body mass index (BMI), and fetal body weight, were strongly associated with the PdE concentration during pregnancy, indicating that PdEs may reflect maternal metabolic adaptation and diagnostic utility to predict adverse pregnancy outcomes at an early stage [119]. Similarly, Elfeky et al. revealed a significant correlation between exosome concentration in maternal circulation and maternal BMI. Specifically, maternal BMI was inversely correlated with the contribution of PdEs to the total exosomes across gestation. A stronger effect was observed in exosomes derived from women of higher BMI in respect to lean, suggesting a potential influence of exosomes on the maternal systemic inflammation during gestation [120]. This study established the exosomal variation could be attributed to maternal BMI.

The role of exosomes derived from adipose tissue (exo-AT) is less investigated in the pathogenesis of GDM. Another study indicates that these exo-AT can function as regulators in the placental glucose metabolism through communication with placenta tissues in GDM, making it a potential to become an effective target for therapeutic intervention to prevent consequences complicated by GDM such as fetal overgrowth [121, 122]. Recently, it has been established that exo-AT might promote insulin resistance (IR) and other obesity-related metabolic statuses in obesity. Novel findings provided the evidence for the pivotal role of the dysregulated release of exo-AT in the onset and development of GDM in obese mothers [122].

Interestingly, PdEs can also function as regulators in the communication with other organs/tissues. Recent studies have identified several exosomal miRs and suggested their potential roles as biomarkers for myogenesis, nutrient metabolism, and muscle mass variation in pathophysiological conditions [123–126]. There may exist a potential link between placenta-specific exosomal miRNAs and skeletal muscle. Nair et al. assessed the concentration of exosomal miRNA in chorionic villi explants derived from 12 pregnancies complicated by GDM compared to 12 normal subjects using next-generation sequencing (NGS) [123]. They further revealed a dysregulated set of 27 placenta-specific exosomal miRNAs and further explored the concentration of several exosomal miRs, including miR-22-3p, miR-125a-3p, miR-197-3p, miR-99b-5p, and miR-224-5p. These specific miRNAs were selected for their differentially expressed patterns between GDM and CTRLs, as well as variation in a consistent pattern in skeletal muscle samples and in GDM maternal circulation. Of note, several differentially expressed miRNAs were predicted to target glucose metabolism-associated genes such as the PI3K/AKT signaling pathway, suggesting their involvement in skeletal muscle insulin sensitivity of GDM. Therefore, placenta-specific exosomal miRNAs might exert a crucial role in the interrelation between gestational tissues and skeletal muscle with subsequent possible effects on peripheral insulin resistance in GDM.

Therefore, such research for the role of exosomes as paracrine vectors might help discover useful research hypotheses and novel knowledge for deciphering GDM pathophysiology and generating valuable and accessible biomarkers for the diagnostics and prediction in GDM. In addition, as regards the dysregulation of miRNA expression which has been linked to the complication of pregnancy, exosomal content including miRNA could be profiled and discovered as biomarkers for GDM. However, their involvement in the pathophysiology of GDM still needs to be further investigated for diagnostic purposes and therapeutic intervention.

### 6.2. Exosomes and miRNAs in GDM Treatment

Exosomes can be potential candidates for effective and regenerative therapies, thus establishing a new therapeutic area in regard to postpartum outcomes of GDM mothers, such as stress urinary incontinence (SUI), a common pathological state observed in nearly 30% of postpartum women [127]. Likewise, therapies based on MSC-exosomes have been also explored and represent as a promising approach in the improvement of GDM-caused myopathy.

Notably, Ni et al. demonstrated that some functional and histological improvements were achieved in a SUI rodent model when treated with hADSCs-exosomes. Additionally, several proteins contained in hADSCs-exosomes were linked to some crucial pathways such as Wnt, PI3K-Akt, and Jak-STAT signaling pathways, which were potentially implicated in skeletal muscle and nerve regeneration [127].

Similarly, Liu et al. reported the capacity of hADSCs-exosomes in increasing type I collagen content through stimulating collagen synthesis and inhibiting collagen degradation in vaginal fibroblasts from SUI women and established promising evidence in the field of therapeutic strategy for treating SUI [128]. Experimental evidence further confirmed the role of exosomes released from fibroblasts of SUI women in regulating endothelial cell angiogenesis [129].

Importantly, it has been established that miRNAs could be a potential candidate for effective and personalized therapy of GDM due to the discovery that exosomes possess diverse functions, including therapeutic function in the GDM avenues.

Moreover, several studies have reported promising approaches in treating GDM. By using microarray analysis, Chen et al. identified differentially expressed genes and miRNAs involved in the regulation of flotillin2 (FLOT2). The results indicated a negative correlation and a target relationship between miR-351 and FLOT2. Specifically, they treated GDM mice with a series of mimic, inhibitor, and small interfering RNA to investigate the bioactivity of miR-351 in insulin resistance (IR), cell apoptosis in pancreatic tissues, and liver gluconeogenesis [130]. The results showed that an upregulation of miR-351 suppressed the expression of FLOT2 with subsequent effects on liver gluconeogenesis by downregulating the PI3K/AKT pathway in GDM mice. These results indicated that miR-351 serve to prevent GDM development, and miR-351 was identified as a therapeutic target in the intervention of GDM.

Another study conducted by Tang et al. explored the role of miR-335-5p on insulin resistance and pancreatic islet *β*-cell secretion via activation of the TGF*β* signaling pathway by downregulating VASH1 expression in GDM mice. They observed that overexpression of miR-335-5p and inhibition of VASH1 might contribute to the downregulation of insulin and insulin release levels [131]. These findings provided evidence for the role of miR-335-5p in the development of insulin resistance and the inhibition of pancreatic islet *β*-cell through downregulating VASH1 and subsequently activating the TGF-*β* pathway in GDM mice, thus providing more clinical insight into the GDM treatment.

## 7. Discussion

Gestational diabetes mellitus is regarded as one of adverse pregnancy complications, presenting an increasing prevalence throughout the world. It may lead to maternal postpartum metabolic disorders, such as obesity and diabetes, and bring about adverse influence on later development of the offspring. Although GDM is well known as a common pregnancy complication, it could not be diagnosed until the late second trimester [6]. Hence, novel biological signatures for timely diagnosis and therapeutic intervention are of significance. Nowadays, early recognition, diagnostic criteria, and therapeutic targets related to GDM are of great interest and with controversies, for diversity exists in race, region, genetics, environmental factors, and diagnostic criteria for GDM [132–135].

It is demonstrated that lifestyle strategy initiated in the first trimester of pregnancy has been proven effective [136–141], reinforcing the importance of exploring biomarkers in early pregnancy. More importantly, identifying novel and available biomarkers in an early pregnancy provides clinical value not only for GDM early diagnosis but also for the prevention of obstetric and maternal-fetal complications.

Our team has investigated thyroid hormone in early pregnancy and revealed a negative correlation between its level and GDM. A low FT4 level in early pregnancy was found to increase the risk for developing GDM [142]. More recently, our team has established an advanced ML model for the early prediction of GDM [143]. Through employing machine learning(ML) models of high accuracy, a clinically cost-effective 7-variable logistic regression (LR) model that achieved effective discriminate power (AUC = 0.77) was ultimately investigated. The results demonstrated that low body mass index (BMI) (≤17) was revealed as a risk factor for GDM. Meanwhile, total 3,3,5′-triiodothyronine (T3) and total thyroxin (T4) showed superiority over free T3 and free T4 in predicting GDM, respectively. Besides, a promising predictive value of lipoprotein was also validated (AUC = 0.66).

As a class of short noncoding RNAs, miRNAs have achieved rising attention in GDM pathophysiology and development. Moreover, miRNAs have also induced interest as mediators of tissue cross-talk, such as adipose tissue and skeletal tissues, in the development of GDM. Notably, apart from previous findings related to miRNA in adipose tissue, adipocyte-derived markers also include adiponectin and leptin [144–146]. Likewise, some other placenta-derived markers, such as follistatin-like-3 [147–149] and placental growth factor [150, 151], could also function as biochemical predictors. These evidences may indicate a potential link of miRNAs to these serum biological signatures, suggesting their capacity as regulators of gene expression at the epigenetic level. Theoretically, the capacity of miRNAs in epigenetic modifications from an early pregnant stage holds evidence for their specific use in predicting GDM. Therefore, further investigation on miRNAs' changes in concentration and corresponding epigenetic alterations in various biological tissues should be carried out.

## 8. Conclusion

In conclusion, we reviewed miRNAs revealed in placental tissues and investigated their roles in metabolic adaptations (e.g., insulin resistance, pancreatic, and *β*-cell function), placental function, and fetal complication. We also reviewed plasma exosomes and molecular content involved in GDM etiology; these evidences help in elucidating GDM pathophysiological pathways. However, their clinically diagnostic and predictive value still needs further investigation. Although several miRs were detected in the first trimester of pregnancy, it is noted that sample collection for miRNA analysis in most studies reviewed were restricted to the late second trimester of gestation. There still exists a lack in the evidence for miRNAs. Therefore, further research is needed in the validation of miRNA profiles for the earlier prediction of GDM. We will conduct more research to establish the potentiality of miRNAs for their predicting value in the diagnosis of GDM later on.

## Figures and Tables

**Table 1:** Studies investigating the regulation of miRNA and related maternal metabolic adaptation in GDM.

miRNA	Regulation	Stage of pregnancy	Source	Cell studied	Putative target	Related metabolic adaptation
miR-222 [55]	↑	38-39 wk	Omental adipose tissue	3T3-L1 cells	ER-*α*	↑estrogen induced insulin resistance
miR-98 [64]	↑	37-40 wk	Placenta	JEG-3 cells	Mecp2, Trpc3	↓insulin-mediated glucose uptake
miR-518d [65]	↑	37-40 wk	Placenta	HEK-293 cells	PPAR*α*	↓glucose intolerance
miR-340 [73]	↑	24-32 wk	Whole blood cells	Lymphocytes	PAIP1	↑maternal fasting insulin
miR-130b, miR-148a [75]	↑	Newborns	HUVECs	HUVECs & BeWo cells	AMPK*α*1	↓glucose metabolism

**Table 2:** Studies investigating the regulation of miRNA and related maternal pancreatic *β*-cell dysfunction in GDM.

miRNA	Regulation	Stage of pregnancy	Source	Cell studied	Putative target	Related pancreatic *β*-cell dysfunction
miR-33a-5p [79]	↑	24-28 wk	Blood samples	INS-1, HEK293T cells	ABCA1	↓cell growth, ↓insulin production
miR-330-3p [80]	↑	24-33 wk	Plasma samples	—	E2F1, CDC42	↓cell proliferation, ↓insulin secretion
miR-494 [83]	↓	—	Peripheral blood	INS-1 cells	PTEN	↓insulin secretion, ↓cell proliferation, ↑cell apoptosis
miR-96 [85]	↓	—	Placental tissue	INS-1, HEK293T cells	PAK1	↓insulin secretion, ↓cell viability
miR-221 [84]	↓	—	Placental tissue of GDM rats	INS-1 cells	PAK1	↓insulin secretion, ↓cell proliferation, ↑cell apoptosis
